# Anatomy and Physiology

**Published:** 1818-02

**Authors:** 


### PART III.

### MONTHLY SKETCH OF FOREIGN MEDICAL SCIENCE.

——— Sparsa Colligens.

### I. ANATOMY AND PHYSIOLOGY.

1. *Anatomy of the Eye.* The ciliary ligament, as our readers will recollect, is a greyish coloured circular band, about two lines in breadth, placed immediately behind the junction of the opaque and transparent corneæ of the eye; and forms a point of intimate and almost inseparable union between the former membrane and the choroid coat. Respecting its structure and functions, no precise knowledge appears hitherto to have been acquired. Montain,* an anatomist of Lyons, is led to consider it as a nerve, possessing close relations with the ganglions, or nerves of organic life. In support of this inference, he asserts, that the ciliary ligament, microscopically examined, displays the same colour, density, and aspect, on rupture and in different modes of division, as the superior cervical ganglion; and that both afford similar results, when treated with the nitric, sulphuric, and muriatic acids, ammonia, and the mercurial solution. With regard to the physiology of the ciliary ligament, he presumes, that it is destined to form the nervous system, and consequently the organ of sensibility, of the iris.†

By the same anatomist, a membrane has been discovered behind the ciliary ligament; to which, from its position, he has given the name of *super-choroidean.* It is very thin, of a brownish colour, four or five lines in breadth, circular, continuous anteriorly with the ciliary ligament, and terminating insensibly between the sclerotic and choroid coats. Its delicate structure, viewed under the microscope, appears particularly cellular and vascular. M. Montain is unable to ascertain its function.

---

* *Mémoire sur plusieurs points d'Anatomie, de Chirurgie, &c. Par Montain jeune, chirurgien en chef de l'hôpital de la Charité de Lyon,* &c. *Bulletin de la Société Médicale d'Emulation,* No. iv. Avril 1817.

† Speaking of the pathology of this ligament, Montain says, that, from various observations and experiments, he is inclined to "attribute to a lesion of this nervous circle the symptoms which frequently accompany the depression of the cataract by the ordinary process." "Indeed (he adds), it is almost impossible, in traversing the sclerotica in this operation, to avoid an injury of the ciliary ligament. Hence the different nervous phænomena, and especially the vomiting, which I have never observed in practising depression by the cornea."

2. *Functions of the respiratory Organs.* From repeated experience of the extreme irritation and distress consequent on the introduction of even a minute portion of extraneous fluid into the human glottis, an inference has been drawn, that very little of such fluid, gaining access into the aërial passages, would suffice to destroy life, by obstructing the necessary communication between the atmosphere and pulmonary blood, or otherwise violently disturbing the respiratory functions. Physiologists have, however, within the last few years, shewn, that a considerable quantity of water, or other simple liquid, may be thrown into the trachea of animals, in the structure and physiology of the thoracic viscera most nearly resembling man, without producing fatal consequences, or even severe or long protracted irritation. But to what extent such injections may be carried with evident impunity, they, who are not acquainted with the recent "experiments upon the introduction of water into the lungs of horses,"* by Professor Gohier, of Lyons, will be little disposed to credit. Into an aperture, made in the trachea of an aged middle-sized horse, were injected, by means of a syringe, in the space of half an hour, about thirty-two *litres* of water before he sank. A little of the fluid was rejected by the tracheal orifice, the mouth and nostril, after each injection. The pulse soon became small and rapid; the body covered with sweat; and progression unsteady. The animal fell and expired. On opening the thorax, the lungs were found heavy and much distended. Little water issued from the bronchiæ; but, on incision of the pulmonary substance, a quantity of the fluid escaped. Another horse, of the same age and size, sustained the injection of forty-two *litres* of water, in consequence of having ejected somewhat more than the former, by coughing. The lungs displayed similar appearances.

Half a *litre* of cold water was injected, on the 4th of March last, through an opening into the trachea of an ass. The animal struggled much to reject the liquid during its introduction; but a few drops only escaped from the wound of the trachea and nostrils. Dyspnœa, hurried motion of the flanks, and wheezing cough, were the symptoms produced. They, however, speedily subsided; and the ass ate and drank as usual during the rest of the day. On the morrow, a *litre*, and on the third day a *litre* and a half, of water were thrown in by the same orifice, without producing any other effects than those observed on the first experiment. The injection of two *litres* of tepid water, on the 7th, was followed by the same symptoms, and efforts to reject it, as on the preceding trials. Respiration continued somewhat difficult during the day; the pulse rose; and a sort of gurgling was, at times, heard in the trachea.

---

* *Expériences sur l'introduction de l'eau dans les poumons des chevaux; par M. Gohier, Professeur à l'école royale vétérinaire de Lyon. Gazette de Santé,* Mai 1817.

The animal coughed, and ate but little. On the following day, all the symptoms had disappeared.

A lean horse, aged about 14, was very little incommoded by the introduction of a *litre* of cold water into an aperture in the trachea, and coughed but once. The injection of two *litres*, on the morrow, induced slight efforts and cough. The motions of the flanks were also, for some hours, hurried. Three *litres** were thrown in the next day, and followed by the same symptoms. The animal died towards night. The lungs displayed nothing peculiar, except some blackish spots, apparently not of recent origin, on their surface. The ventricles of the heart contained very black blood. The death of the horse appeared to be rather attributable to weakness than to the effects of the experiment,

Professor Gohier, and Montegre, Editor of the Journal from which these experiments have been transcribed, are willing to infer from them the practicability of introducing medicated fluids into the lungs of animals, whereby the progress of the more commonly destructive forms of pulmonary disease may be arrested or controlled; and thus speculate on the re-application of the powerful mean of bronchial injection to human and veterinary medicine. Montegre, moreover, thinks, that these "singular experiments" throw light upon the curious fact of respiration of the fœtus amid the fluid of the amnios, recently demonstrated by the anatomist Béclard.†

* The French *litre*, we may here remind our readers, is somewhat more than equivalent to two English wine pints. Edit.

† See *Medico-Chirurgical Journal and Review*, vol. i. page 269.

